# Hyperammonaemic encephalopathy: a remarkably rare complication after bariatric surgery

**DOI:** 10.1093/jscr/rjad227

**Published:** 2023-05-02

**Authors:** Shaurya Jhamb, Tejminder S Sidhu, Scott Whiting

**Affiliations:** Department of Surgery, Townsville University Hospital, Townsville, Australia; Department of Surgery, Townsville University Hospital, Townsville, Australia; Department of Surgery, Townsville University Hospital, Townsville, Australia

**Keywords:** Hyperammonaemia, Urea cycle, Bariatric surgery, encephalopathy, hepatic, gastric sleeve

## Abstract

Hyperammonaemia is a metabolic disorder with elevated blood ammonia levels. Here we describe a case of hyperammonaemia associated encephalopathy as an incredibly rare, potentially fatal and treatable complication associated with bariatric surgery. This case highlights the importance of longer-term follow-up after bariatric surgery.

## INTRODUCTION

**Figure 1:**
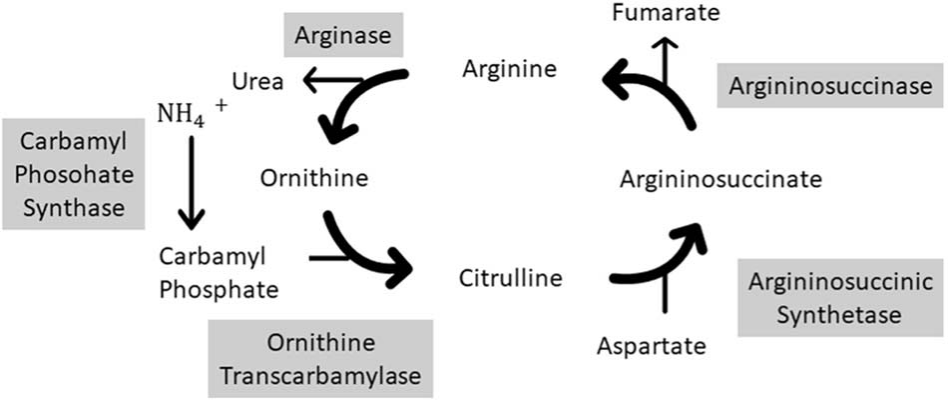
Urea cycle metabolism—biochemical reactions forming Urea from Ammonia [1].

Hyperammonemia is a metabolic abnormality characterized by elevated levels of ammonia in the blood. Hyperammonemic encephalopathy is an incredibly rare and under recognized complication of bariatric surgery with high morbidity and mortality [1, 2]. With less than 25 reported cases in the literature there are several postulated theories and pathophysiological mechanisms linking hyperammonaemia and bariatric surgery [3]. In the context of bariatric surgery patients, it is presumed to occur as a result of complex metabolic pathways unique to these patients that can be unveiled in states of malnutrition [2]. There appears to be a combination of factors that cause malnourished Roux-en-Y Gastric Bypass (RYGB) patients to be shunted down ammonia producing pathways. In addition to genetic factors, non-genetic factors including increased nitrogen waste and microbiome changes precipitated by the new anatomy are predicted to play a role [1]. RYGB related hyperammonaemic encephalopathy has a reported fatality of 50% and can occur at variable time intervals after a RYGB with reports from 1 month to 28 years after surgery [3]. Ammonia is a by-product of amino acid degradation that occurs in the liver (See Fig. 1). Disruption of this process can lead to a build-up of blood ammonia levels [4].

Hyperammonaemic encephalopathy is usually a condition related to severe liver disease resulting in increased production of ammonia or conversely from decreased elimination of ammonia (see Fig. 1) in conditions related to congenital errors of metabolism, congenital portosystemic shunts or intake of certain drugs [1, 5].

High levels of ammonia are toxic to the brain and as a result the signs and symptoms are predominantly neurological. Acute hyperammonaemia symptoms can include vomiting, lethargy, drowsiness, seizures, multiorgan failure and coma. Chronic hyperammonaemia can present with headaches, cognitive deficits, behavioural changes, tremors, ataxia and seizures [6]. The exact mechanism of this complication is still poorly understood. Mortality from this complication is significant; early recognition, diagnosis and intervention is pivotal to improving outcomes [2].

### CASE REPORT

A 49-year-old female with a background history of elective sleeve gastrectomy and conversion to RYGB presented with a chronic recurrent small bowel obstruction (SBO). Immediately preceding the current presentation, this patient had been suffering from increasingly frequent bowel obstructions causing a malnourished state. Relevant medical history included paroxysmal Atrial Fibrillation, multiple sclerosis and possible liver cirrhosis secondary to alcohol and non-alcoholic steatohepatitis with associated oesophageal varices noted on Computer Tomography (CT) scans. She had recurrent bowel obstruction symptoms for 18–24 months and was initially managed conservatively with decompression on this admission, with partial resolution of symptoms.

**Figure 2:**
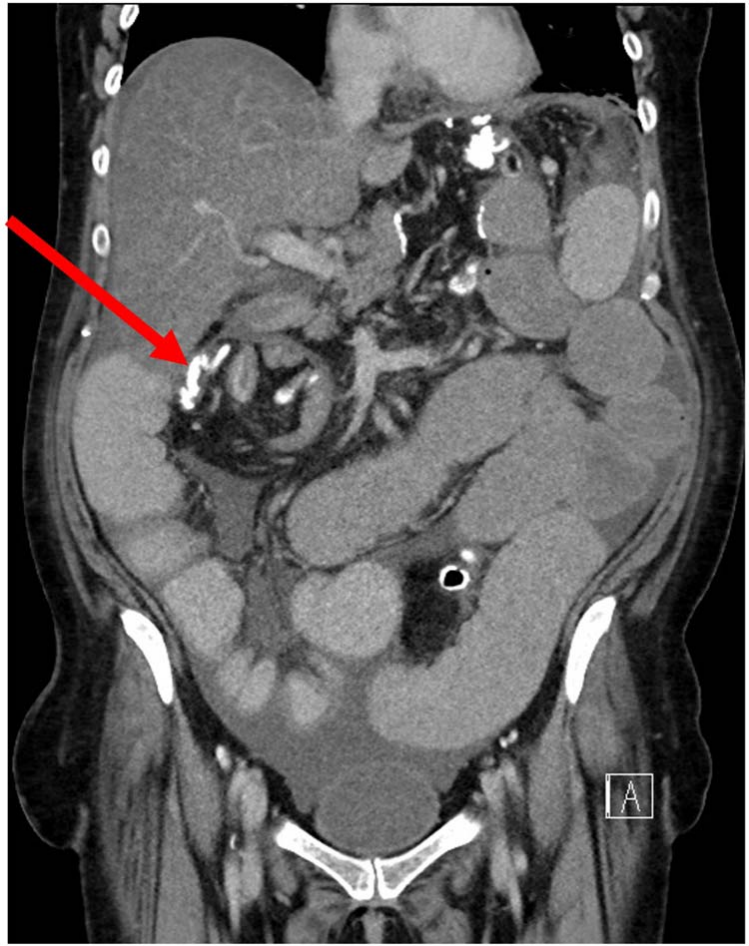
Coronal CT of SBO secondary to internal hernia.

**Figure 3:**
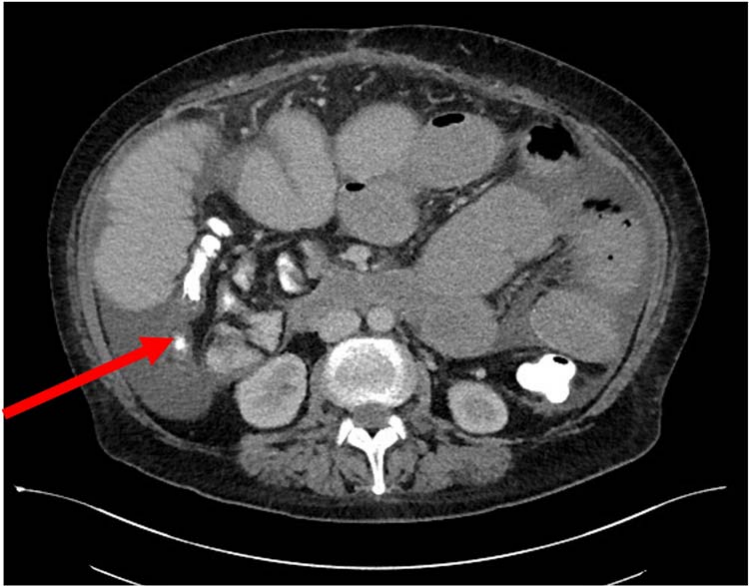
Axial CT of SBO secondary to internal hernia.

**Figure 4:**
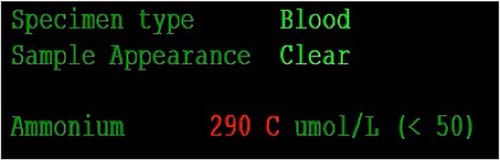
Blood ammonium level prior to dialysis.

Her case was reviewed by a bariatric surgeon, who noted an internal hernia on CT causing the recurrent obstruction and subsequently planned for surgery (see Figs 2 and 3). On day 6, at the time of consenting for the procedure, she had a rapid neurological deterioration with fluctuating levels of consciousness (GCS10- E4V1M5), complete aphasia, bilaterally dilated pupils (9–10 mm), increased tone, hyper-reflexia and sustained clonus of 10+ beats in lower limbs. A broad list of differentials was considered due to her medical history. CT (Brain, head and neck angiogram and Venogram) scans reported no acute intracranial pathology, plasma ammonia level was 290 μmol/L (<50 μmol/L) (see Fig. 4), C-reactive protein (CRP) 44 mg/L (<10 mg/L) and ketosis with no acidosis.

Over the next 48 h, she had fluctuating levels of consciousness, seizure-like episodes and had strong hepatic fetor. She was reviewed by multiple medical teams including Gastroenterology and Endocrinology who presumed this to be a presentation of hepatic encephalopathy. However, there was a high degree of suspicion for her symptoms to be a result of hyperammonaemic encephalopathy and she was treated accordingly:

- Transferred to the Intensive Care Unit (ICU) for ongoing investigation, management and airway protection.
- Commenced on parenteral nutrition.
- Haemofiltration to rapidly reduced ammonia levels.
- Commencement of ammonia-scavenging drugs.

Due to her complex medical history, there was a delay in recognizing her diagnosis as hyperammonemic encephalopathy due to chronic malnutrition, chronic liver disease and prolonged fasting during her multiple admissions. Her neurological symptoms resolved after the aforementioned management was undertaken. She went on to have her internal hernia successfully closed for definitive management.

## DISCUSSION

This diagnostic challenge also highlighted the importance of having a high index of suspicion for these rarer complications in post bariatric surgery patients. Key strategies to supportive management hyperammonaemic encephalopathy included:

- Advocating for further investigations
- Escalating cares to ICU
- Early initiation of Total Parenteral Nutrition (TPN)
- Nutritional supplementation
- Initiating dialysis
- Ammonia-scavenging drugs

It is also noteworthy that the internal defect which caused the recurrent SBOs more commonly occurs where defect closures are not routine. In patients who have travelled for bariatric surgery, closure of created internal defects should be mandatory. This presentation also draws attention to the importance of regular longer-term follow-up of patients with bariatric surgeons and dieticians.

## CONCLUSION

Hyperammonemia encephalopathy is a remarkably rare, potentially fatal and treatable complication associated with bariatric surgery. This case highlights the importance of longer-term follow-up after bariatric surgery.
